# Increasing basal nitrogen fertilizer rate improves grain yield, quality and 2-acetyl-1-pyrroline in rice under wheat straw returning

**DOI:** 10.3389/fpls.2022.1099751

**Published:** 2023-01-12

**Authors:** Jun Wang, Yuanyuan Qiu, Xingyu Zhang, Zhou Zhou, Xian Han, Yang Zhou, Li Qin, Kun Liu, Siyu Li, Weilu Wang, Yun Chen, Jianchang Yang, Lijun Liu

**Affiliations:** ^1^Jiangsu Key Laboratory of Crop Genetics and Physiology/Jiangsu Co-Innovation Centre for Modern Production Technology of Grain Crops, Yangzhou University, Yangzhou, China; ^2^Joint International Research Laboratory of Agriculture and Agri-Product Safety, the Ministry of Education of China, Yangzhou University, Yangzhou, Jiangsu, China; ^3^College of Bioscience and Biotechnology, Yangzhou University, Yangzhou, Jiangsu, China

**Keywords:** wheat straw returning, rice yield, grain quality, 2-acetl-1-pyrroline, basal nitrogen fertilizer rate

## Abstract

Straw returning plays an essential role in crop yields and the sustainable development of agriculture. However, the effects and mechanisms of nitrogen (N) fertilizer management on grain yield, quality and aroma substance 2-acetyl-1-pyrroline (2-AP) content under wheat straw returning are still unclear. In this field experiment, two *japonica* rice cultivars were used as materials, wheat straw non-returning (NS) and wheat straw full returning (WS) were designed coupled with two N application ratios, namely basal fertilizer: tiller fertilizer: panicle fertilizer =5:1:4 (local farmers’ fertilizer practice, LFP) and 7:1:2 (increasing basal fertilizer rate, IBF) under the total N application rate of 270 kg ha^-1^. The effects of the four treatment combinations (NS-LFP, NS-IBF, WS-LFP, WS-IBF) on yield, cooking and eating quality, and 2-AP content in rice were investigated. The two-year (2020, 2021) results showed that: 1) WS-IBF significantly increased the number of panicles and grains per panicle, leading to the increase in grain yield by 6.67%–12.21%, when compared with NS-LFP, NS-IBF and WS-LFP. 2) WS-IBF enhanced the taste value, peak viscosity, breakdown value, the ratio of amylopectin to amylose, and the ratio of glutelin to prolamin while reducing the setback value and amylose content of rice flour. 3) Compared with NS, WS increased the activities of proline dehydrogenase and ornithine transaminase, the synthetic precursors of 2-AP, and finally increased 2-AP content in rice grains. WS-IBF slightly decreased 2-AP content, but there was no significant difference with WS-LFP. The above results indicated that adjusting the N regime and increasing basal N fertilizer rate under wheat straw returning is conducive to improving grain yield, cooking and eating quality, and 2-AP content in rice.

## 1 Introduction

Crop straw is an important biomass resource in agricultural production and a major source of soil organic matter. Straw returning plays a crucial role in improving soil aggregate structure, enhancing soil aeration and hydrophobicity, increasing soil nutrients, and promoting crop yield (Zhao et al., 2016; Berhane et al., 2020). China is the world’s largest straw producer, accounting for 20%-30% of the global output. The fertilizer utilization rate of nearly 700 million tons of collectible straw resources each year reaches 53.9%, of which 39% are directly returned to the field (Li et al., 2018). Among the three major categories of crop straw resources, corn, rice and wheat, wheat straw returning accounted for the highest percentage of collectible straw, reaching 73.4% (Shi et al., 2017). A meta-analysis revealed that among 142 straw-returning experiments in China, nearly 92% of the experimental results showed that straw returning has a vital effect on improving soil properties and increasing crop yield (Zhao et al., 2015).

The middle-lower reaches of the Yangtze River are China’s main rice-wheat rotation areas. Straw burning used to be the simplest and most convenient method in this area due to tight crop rotation and short agricultural operation time. With the promotion of the straw mechanization return policy, the mechanized rice cultivation technology of returning wheat straw to the field gradually formed, but in the actual cultivation, farmers still follow the traditional field fertilization management methods, resulting in the phenomenon of rice runt-seedling (Hofmeier et al, 2015).

Previous studies have shown that the effect of straw returning on rice growth is closely related to straw type, returning method, tillage system, soil texture and nitrogen fertilizer application, etc (Zhang et al., 2015; Bu et al., 2020; Shan et al., 2021). However, it is mostly believed that under straw returning, increasing basal N fertilizer application can effectively counteract the insufficient amount of rice population in the early growth period and the competition for nitrogen during the peak of straw decomposition (Grzyb et al., 2021; Liu et al., 2021). Cui et al. (2020) suggested that soil microorganisms are stimulated by the imbalance of C-N measurement during the decomposition of straw, which leads to faster decomposition of sedimentary organic carbon by microorganisms and promotes the increase of nitrogen content of light fraction organic carbon and the mineralization of heavy fraction nitrogen in the soil, thereby increasing crop yield (Dong et al., 2019). Wu et al. (2021) and Chen et al. (2017) found that straw returning can improve yield by increasing dry matter accumulation and photosynthetic characteristics of rice, and can significantly improve rice taste value, breakdown value and protein content, while increasing basal fertilizer rate can further improve rice cooking and eating quality. The change of soil carbon to nitrogen ratio affects the supply of carbon and nitrogen material for grain filling at maturity. With the increase of nitrogen application ratio in the early stage, the head milled rice, chalky grain rate, protein content and setback value gradually decreased, while the peak viscosity, breakdown value and gel consistency increased (Wan et al., 2006). Meanwhile, the nutrients released by straw decay will prolong the functional period of the upper three leaves in the late stage of rice growth, enhance the function of photosynthetic and transport systems during the filling stage, and promote the development of glumes and improve the appearance and taste quality of rice (Wu et al., 2021).

Rice aroma is an important economic evaluation indicator of rice quality. 2-acetyl-1-pyrroline (2-AP) is the main contributor to rice aroma characteristic compounds, and its concentration difference is the main reason for the strength of aroma between varieties (Widjaja et al., 1996). Numerous studies have shown that the synthesis of 2-AP is mainly based on three precursors: proline, glutamate and ornithine which are used as nitrogen sources catalyzed by proline dehydrogenase, pyrroline-5-carboxylic acid synthetase and ornithine transaminase to form pyrroline-5-carboxylic acid, subsequently decarboxylated by a pyrroline-5-carboxylate decarboxylase to 1-pyrroline and then combined with acetyl coenzyme A to synthesize 2-AP with the participation of pyrroline acetyltransferase (Huang et al., 2008; Okpala et al., 2019). The accumulation of 2-AP in rice is mainly synthesized in the plant’s shoots. Two mechanisms have been found for the accumulation of 2-AP in the grains. One is that 2-AP is synthesized in leaves and stem sheaths and then transported to the grains; the other suggests that proline is transported from leaves to grains, where 2-AP is synthesized (Wei et al., 2017). In addition to the genetic characteristics of rice varieties, the soil environment is also an essential factor affecting the synthesis of 2-AP in rice. Yang et al. (2012) found that the aroma and yield of fragrant rice are significantly enhanced in soils rich in organic matter and high total nitrogen content, while Singh et al. (2003) concluded that fragrant rice aroma is negatively correlated with soil nitrogen content. There are few reports on the effect of wheat straw returning on 2-AP in rice. Previous studies have shown that leguminous green manure return can increase soil organic matter and total nitrogen content, thereby increasing rice free-proline content and promoting 2-AP synthesis in grain (Song et al., 2022). The effects of the N fertilizer application period and rate on 2-AP synthesis were also different. Mo et al. (2019) found that increasing the N fertilizer rate at the booting stage and maintaining soil water potential at -25 ± 5kPa can significantly increase the synthesis of amino acids, thereby increasing the content of rice aroma substances. Li et al. (2022) suggested that water and nitrogen management at the tillering stage is an important factor affecting 2-AP synthesis, and increasing the proportion of nitrogen fertilizer application at the tillering stage can promote 2-AP synthetase activity in grains so as to improve the yield and aroma of fragrant rice.

Wheat straw returning is the most important conservation tillage method in the rice-wheat rotation area (Jin et al, 2020). However, the effects of coupling of wheat straw returning and N regime on cooking and eating quality and aroma substances in rice have rarely been reported. In this paper, two *japonica* cultivars were used as materials, and two nitrogen application ratios were designed under wheat straw returning or not returning in field conditions. The objectives were to 1) investigate the coupling effects of wheat straw returning and N regime on grain yield and quality; 2) illuminate the changes in 2-AP content in grains and their physiological mechanisms.

## 2 Materials and methods

### 2.1 Experimental site

The field experiment was conducted at the experimental farm of Jiangsu Key Laboratory of Crop Cultivation and Physiology, Yangzhou University, Jiangsu Province, China (32° 30′ N, 119° 25′ E) during the rice-growing seasons of 2020 and 2021. The previous crop was wheat, and the soil texture was sandy loam consisting of 21.3 g kg^-1^ organic matter, 1.32 g kg^-1^ total nitrogen, 33.5 mg kg^-1^ available phosphorus and 89.7 mg kg^-1^ available potassium.

### 2.2 Experimental design

Two *japonica* rice, Nanjing 9108 (NJ9108) and Huaidao 5 (HD5) were used as materials. A two-factor randomized block design was adopted in this experiment. The first factor was two wheat straw returning treatments. One was wheat straw non-returning (NS), and the other was wheat straw full returning (WS). In the WS treatment, the wheat straw was cut into 5 cm long pieces after the previous wheat crop was harvested and then rototilled into the top 20 cm soil layer one week before rice transplanting. The second factor was two N application ratio treatment. The total N application during the rice growth period was 270 kg ha^-1^, which was the common N rate for the local rice farmers(Yang et al, 2022; Liu et al., 2022). One treatment was the local farmers’ fertilizer practice (LFP), and the ratios of basal fertilizer, tiller fertilizer and panicle fertilizer to the total N rate were 50%, 10% and 40%. The other was increasing basal fertilizer rate (IBF), and the ratios of basal fertilizer, tiller fertilizer and panicle fertilizer to the total N rate were 70%, 10% and 20%. There were four treatment combinations, i.e., NS-LFP, NS-IBF, WS-LFP and WS-IBF.

Across both years, seedlings were sown on May 15-16 in 2020 and 2021 raised in a seedbed, and manually transplanted on June 12-13 at a hill spacing of 13.5cm× 25 cm. The plot area was 20 m^2^ arranged in random blocks and repeated three times. N (urea) fertilizer was broadcasted at pre-transplanting (1 day before transplanting), tillering (7 days after transplanting) and panicle initiation (the first appearance of a differentiated apex). Before transplanting, P_2_O_5_ and K_2_O were broadcasted as basal fertilizers at 40.5 kg ha^−1^ and 125 kg ha^−1^, respectively.

### 2.3 Measurements and methods

#### 2.3.1 Tillering dynamics

Ten hills of rice plants were selected continuously as an observation point in each plot, and the tiller numbers were observed at the mid-tillering stage, panicle-initiation stage, heading stage and maturity stage.

#### 2.3.2 Soil total nitrogen content

Soil samples were collected at the mid-tillering stage, panicle-initiation stage, heading stage and maturity stage from five soil cores (0-20cm soil depth) in each plot. After removing the plant residues, they were mixed evenly, then placed in a ventilated place for natural air drying. After grinding, soil samples were screened with 60 mesh and the total nitrogen (TN) content was determined by the Kjeldahl method (Bao, 2000).

#### 2.3.3 Grain yield and dry matter

Grain yield was determined from a harvest area of 5.0 m^2^ in each plot and adjusted to 14% moisture, and these grain samples were retained for quality determination. The panicle number per square meter was calculated by counting the panicle numbers of 50 hills of rice randomly selected from each plot (excluding the border areas). According to the average number of panicles, representative samples of 6 hills of rice from each plot were collected to calculate the number of grains per panicle, seed-setting rate and grain weight.

Six hills of rice were sampled in each plot at the mid-tillering stage, panicle-initiation stage, heading stage and maturity stage. The roots and aboveground parts were separated, washed and drained then put into the oven. After removing water at 105°C for 30 min, then dried at 70°C to constant weight for weighing.

#### 2.3.4 Grain quality

The grains were air-dried and stored for three months, the gel consistency, amylose content and amylopectin content were determined with reference to (GB/T17891-2017 High-Quality Rice Grain). The protein and its components content of grains were measured with milled rice as the test sample using an automatic Kjeldahl apparatus, wherein the protein components were measured according to the method of Tang, (1999), the albumin was extracted with distilled water, globulin was extracted with 0.06 mol L^-1^ NaCl solution, prolamin was extracted with 75% ethanol solution, and glutenin was extracted with 0.04 mol L^-1^ NaOH solution, and the above extracts were tested to determine the protein components content.

The cooking and eating quality were measured by using the Japanese cooking rice taste meter STA1B to determine the rice characteristics and its related taste properties with Heilongjiang japonica rice in China as the reference standard. The RVA spectral characteristic value (RVA value) was measured with 3 g of a 100-mesh sieved rice flour sample, plus 25 g of ultrapure water, using a rapid viscosity analyzer (Model 3D, Newport Scientific, Australia) according to AACC (American Association of Cereal Chemists) procedure for rapid determination, and its supporting software TWC (Thermal cycle for windows) was used for data analysis.

#### 2.3.5 2-AP content and enzyme activities

Fresh samples of rice leaves and grains were taken at 10-day intervals from heading to maturity, frozen in liquid nitrogen, fully ground with a mixing ball mill (RETSCH MM400, Germany) and stored in a -70°C refrigerator. The 2-AP content in grains was determined using the method of Yang et al. (2022), the ground grain samples (0.8g) were extracted with 3ml of pure dichloromethane using an ultrasonic machine for 3h, then the supernatant was detected by GC-MS (GCMS-6890-5973, Agilent, USA). The proline content was determined following the Banu et al. (2010) method, the proline dehydrogenase (PDH) and ornithine aminotransferase (OAT) activities were determined according to the method of Ncube et al. (2013) and Umair et al. (2011), respectively. The 2-AP contents were expressed as ng g^-1^; the proline contents were expressed as μg g^−1^; the PDH and OAT activities were expressed as μmol g^-1^ h^-1^ FW.

### 2.4 Data analysis

Experimental data were analyzed using SPSS version 28, and the mean value was tested by least significant difference (LSD), P=0.05. Plots were made using Origin 2021.

## 3 Results

### 3.1 Yield and yield components

Two-year experiments showed that WS-IBF significantly increased the number of panicles and grains per panicle, resulting in a substantial increase in yield but a greater decrease in seed-setting rate (**Table 1**). The yield of WS-IBF increased by 6.7–9.0% and 10.3–12.2% compared with WS-LFP and NS-LFP treatments. Compared with NS-LFP treatment, NS-IBF treatment promoted the occurrence of rice tillers in the early stage (**Table S1**) and increased the number of panicles, but significantly reduced the percentage of productive tiller, number of grains per panicle, grain weight and seed-setting rate, resulting in a yield decrease of 8.3–12.7%. Based on conventional nitrogen proportional treatment (NS-LFP), wheat straw returning (WS-LFP) reduced the number of panicles, but would significantly increase the number of grains per panicle and grain weight, resulting in a slight increase in yield, but the difference between the two was not significant.

**Table 1:** Grain yield and its component factors.

Time	Cultivar	Treatment	Panicle number (×10^4^ ha^-1^)	Grains number per panicle	Grain weight (mg)	Seed-setting rate (%)	Grain yield (t ha^-1^)
2020	NJ9108	NS-LFP	290.50 ± 2.88 c	147.67 ± 2.42 c	25.41 ± 0.11 b	87.19 ± 0.12 a	9.50 ± 0.20 b
		NS-IBF	296.50 ± 4.04 b	130.50 ± 2.43 d	25.12 ± 0.17 c	85.25 ± 0.40 b	8.29 ± 0.27 c
		WS-LFP	280.67 ± 3.67 d	155.82 ± 2.56 b	25.75 ± 0.06 a	86.83 ± 0.20 a	9.78 ± 0.19 b
		WS-IBF	307.00 ± 3.69 a	161.00 ± 3.90 a	25.59 ± 0.09 a	84.25 ± 0.16 c	10.66 ± 0.29 a
	HD5	NS-LFP	281.67 ± 1.90 c	141.00 ± 2.83 c	25.71 ± 0.14 b	86.60 ± 0.20 a	8.84 ± 0.24 b
		NS-IBF	289.33 ± 3.20 b	134.54 ± 2.36 d	25.09 ± 0.23 c	83.49 ± 0.37 b	8.11 ± 0.21 c
		WS-LFP	268.67 ± 2.42 d	149.00 ± 3.29 b	26.29 ± 0.14 a	86.22 ± 0.12 a	9.07 ± 0.28 b
		WS-IBF	295.33 ± 2.66 a	154.00 ± 3.10 a	26.05 ± 0.15 a	83.16 ± 0.56 b	9.85 ± 0.28 a
2021	NJ9108	NS-LFP	285.17 ± 2.93 c	148.00 ± 1.79 c	25.36 ± 0.07 b	88.01 ± 0.37 a	9.42 ± 0.22 b
		NS-IBF	291.00 ± 2.76 b	137.00 ± 1.90 d	24.77 ± 0.12 c	85.05 ± 0.39 c	8.40 ± 0.19 c
		WS-LFP	270.50 ± 3.83 d	160.35 ± 3.56 b	25.81 ± 0.14 a	86.64 ± 0.50 b	9.70 ± 0.19 b
		WS-IBF	300.33 ± 2.64 a	166.50 ± 1.87 a	25.60 ± 0.18 a	82.06 ± 0.26 d	10.50 ± 0.15 a
	HD5	NS-LFP	278.00 ± 2.68 c	139.00 ± 3.29 c	26.17 ± 0.18 b	86.09 ± 0.47 a	8.70 ± 0.19 b
		NS-IBF	285.00 ± 3.10 b	131.21 ± 2.20 d	25.42 ± 0.14 c	83.11 ± 0.30 c	7.90 ± 0.10 c
		WS-LFP	271.17 ± 2.14 d	148.67 ± 1.86 b	26.51 ± 0.17 a	84.23 ± 0.22 b	9.00 ± 0.12 b
		WS-IBF	292.50 ± 3.01 a	153.48 ± 3.34 a	26.31 ± 0.20 ab	81.30 ± 0.54 d	9.60 ± 0.33 a
Source of variation							
Year (Y)			53.285**	5.610*	14.780**	127.278**	6.395*
Cultivar (C)			148.013**	155.549**	287.281**	370.509**	196.853**
Treatment (T)			318.322**	398.872**	195.363**	694.163**	317.609**
Y×C			15.368**	26.366**	43.123**	26.848**	NS
Y×T			NS	NS	NS	44.428**	NS
C×T			NS	13.777**	5.147**	7.435**	6.272**
Y×C×T			4.705**	NS	3.702*	11.705**	NS

NS-LFP, wheat straw non-returning and the ratios of basal fertilizer: tiller fertilizer: panicle fertilizer=5:1:4; NS-IBF, wheat straw non-returning and the ratios of basal fertilize: tiller fertilizer: panicle fertilizer=7:1:2; WS-LFP, wheat straw full returning and the ratios of basal fertilizer: tiller fertilizer: panicle fertilizer=5:1:4; WS-IBF, wheat straw full returning and the ratios of basal fertilize: tiller fertilizer: panicle fertilizer=7:1:2. Different lowercase letters in the same column indicate statistical significance at P < 0.05 in the same variety in the same year. * and ** significant at P < 0.05 and P < 0.01, respectively; NS, nonsignificant at P < 0.05 level.

### 3.2 Dry matter weight in root and shoot

WS-IBF treatment had the highest root and shoot dry matter weight at all stages except the mid-tillering stage (**Table 2**). Under the same N fertilizer ratio, WS treatments reduced root and shoot dry matter by 2.9–17.9% and 2.2–16.4% at the mid-tillering stage, respectively, compared with NS treatments, but significantly increased dry matter weight from the panicle initiation stage to the maturity stage. Under the IBF ratio, WS-IBF treatment could dramatically improve root and shoot dry matter weight at the maturity stage. Under the LFP ratio, WS-LFP treatment had no significant increase in dry matter weight compared with NS-LFP treatment, which was consistent with the yield performance.

**Table 2:** Dry matter weight in root and shoot of rice (t ha^-1^).

Time	Cultivar	Treatment	Mid-tillering		Panicle-initiation		Heading		Maturity	
			Root	Shoot	Root	Shoot	Root	Shoot	Root	Shoot
2020	NJ9108	NS-LFP	0.32 ± 0.03 ab	1.68 ± 0.07 ab	0.72 ± 0.06 b	5.52 ± 0.22 b	1.25 ± 0.07 bc	11.24 ± 0.20 b	1.12 ± 0.07 bc	19.55 ± 0.13 b
		NS-IBF	0.38 ± 0.06 a	1.81 ± 0.09 a	0.79 ± 0.04 b	5.65 ± 0.16 b	1.11 ± 0.13 c	10.43 ± 0.31 c	0.90 ± 0.09 c	18.34 ± 0.30 c
		WS-LFP	0.28 ± 0.02 c	1.46 ± 0.06 b	0.84 ± 0.09 ab	6.17 ± 0.20 ab	1.38 ± 0.08 ab	11.72 ± 0.24 b	1.27 ± 0.10 ab	20.26 ± 0.44 b
		WS-IBF	0.36 ± 0.02 ab	1.77 ± 0.04 a	0.98 ± 0.04 a	6.82 ± 0.32 a	1.50 ± 0.04 a	12.36 ± 0.07 a	1.39 ± 0.09 a	21.43 ± 0.28 a
	HD5	NS-LFP	0.29 ± 0.03 ab	1.51 ± 0.12 ab	0.60 ± 0.07 b	4.76 ± 0.12 c	1.21 ± 0.06 b	10.53 ± 0.51 bc	1.10 ± 0.02 b	18.79 ± 0.19 b
		NS-IBF	0.34 ± 0.05 a	1.74 ± 0.06 a	0.67 ± 0.05 ab	5.34 ± 0.22 b	0.84 ± 0.03 c	9.74 ± 0.15 c	0.78 ± 0.05 c	17.74 ± 0.24 c
		WS-LFP	0.25 ± 0.02 b	1.42 ± 0.09 b	0.73 ± 0.03 ab	5.70 ± 0.25 b	1.29 ± 0.07 ab	11.06 ± 0.18 ab	1.18 ± 0.08 ab	19.21 ± 0.26 b
		WS-IBF	0.33 ± 0.04 ab	1.68 ± 0.10 ab	0.85 ± 0.09 a	6.23 ± 0.13 a	1.38 ± 0.05 a	11.55 ± 0.14 a	1.26 ± 0.02 a	20.34 ± 0.35 a
2021	NJ9108	NS-LFP	0.28 ± 0.04 ab	1.59 ± 0.15 ab	0.69 ± 0.06 b	5.19 ± 0.33 b	1.16 ± 0.05 bc	10.97 ± 0.11 bc	1.08 ± 0.07 bc	19.38 ± 0.48 b
		NS-IBF	0.36 ± 0.03 a	1.80 ± 0.13 a	0.71 ± 0.05 b	5.67 ± 0.12 b	1.00 ± 0.08 c	10.16 ± 0.26 c	0.86 ± 0.08 c	17.92 ± 0.32 c
		WS-LFP	0.23 ± 0.04 b	1.33 ± 0.05 b	0.78 ± 0.03 b	6.05 ± 0.74 ab	1.33 ± 0.06 ab	11.44 ± 0.47 ab	1.20 ± 0.12 ab	19.95 ± 0.20 b
		WS-IBF	0.34 ± 0.02 a	1.74 ± 0.10 a	1.02 ± 0.06 a	7.20 ± 0.21 a	1.46 ± 0.09 a	12.09 ± 0.28 a	1.34 ± 0.06 a	21.01 ± 0.25 a
	HD5	NS-LFP	0.23 ± 0.04 bc	1.37 ± 0.07 b	0.56 ± 0.06 b	4.47 ± 0.18 c	1.05 ± 0.10 bc	10.35 ± 0.74 bc	0.91 ± 0.04 bc	18.44 ± 0.28 b
		NS-IBF	0.37 ± 0.06 a	1.70 ± 0.10 a	0.62 ± 0.05 b	5.13 ± 0.40 bc	0.85 ± 0.13 c	9.61 ± 0.56 c	0.70 ± 0.12 c	17.08 ± 0.15 c
		WS-LFP	0.19 ± 0.02 c	1.21 ± 0.08 b	0.69 ± 0.07 ab	5.34 ± 0.16 b	1.21 ± 0.11 ab	11.15 ± 0.27 ab	1.03 ± 0.10 ab	18.90 ± 0.45 ab
		WS-IBF	0.32 ± 0.05 ab	1.65 ± 0.07 a	0.82 ± 0.05 a	6.09 ± 0.15 a	1.35 ± 0.07 a	11.85 ± 0.10 a	1.22 ± 0.10 a	20.07 ± 0.74 a
Source of variation										
Year (Y)			NS	6.654*	NS	NS	6.082*	NS	7.853*	10.279**
Cultivar (C)			4.728*	11.807**	37.805**	41.694**	18.754**	23.071**	17.752**	58.470**
Treatment (T)			17.213**	32.450**	34.545**	44.655**	50.419**	49.251**	52.895**	108.772**
Y×C			NS	NS	NS	NS	NS	NS	NS	NS
Y×T			NS	NS	NS	NS	NS	NS	NS	NS
C×T			NS	NS	NS	NS	NS	NS	NS	NS
Y×C×T			NS	NS	NS	NS	NS	NS	NS	NS

NS-LFP, wheat straw non-returning and the ratios of basal fertilizer: tiller fertilizer: panicle fertilizer=5:1:4; NS-IBF, wheat straw non-returning and the ratios of basal fertilizer: tiller fertilizer: panicle fertilizer=7:1:2; WS-LFP, wheat straw full returning and the ratios of basal fertilizer: tiller fertilizer: panicle fertilizer=5:1:4; WS-IBF, wheat straw full returning and the ratios of basal fertilizer: tiller fertilizer: panicle fertilizer=7:1:2. Different lowercase letters in the same column indicate statistical significance at P < 0.05 in the same variety in the same year. * and ** significant at P < 0.05 and P < 0.01, respectively; NS, nonsignificant at P < 0.05 level.

Under the condition of WS, WS-IBF treatment promoted dry matter weight in the whole rice growth period compared with WS-LFP treatment, while under the NS, NS-IBF treatment only increased dry matter weight from the mid-tillering stage to the panicle-initiation stage. Compared with NS-LFP treatment, NS-IBF treatment reduced root and shoot dry matter weight by 19.8–29.7% and 5.6–7.4% at the maturity stage, respectively. In contrast, WS-IBF treatment increased root and shoot dry matter weight at the maturity stage by 6.8–18.5% and 5.3–6.2%, respectively, compared with WS-LFP treatment.

### 3.3 Soil total nitrogen content

There was no significant difference in soil TN content among varieties at each period, but the difference between treatments was extremely significant (**Table S2**). IBF treatments increased soil TN content at the mid-tillering stage, while LFP treatments increased soil TN content at the middle and late stages. Compared with NS treatments, WS treatments significantly increased soil TN content from panicle-initiation stage to maturity stage.

### 3.4 Cooking and eating quality, RVA profiles and amylose content of grains

**Figure 1:**
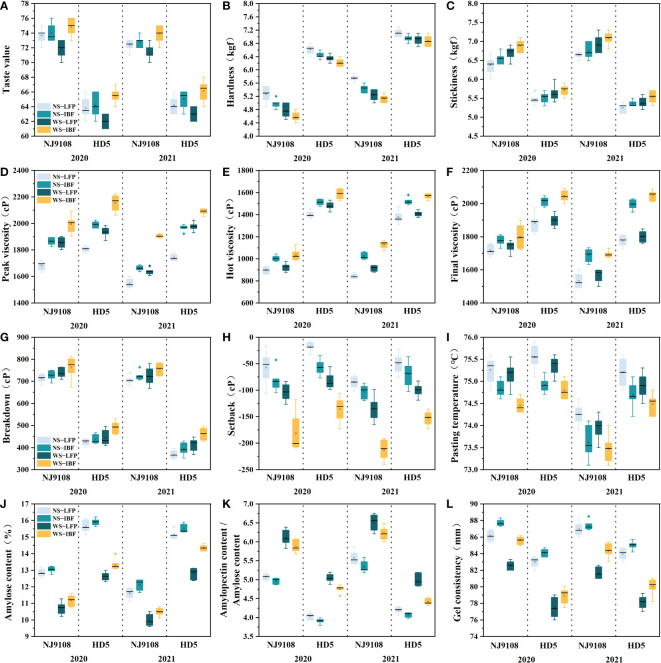
Cooking and eating quality, RVA profiles and amylose content of grains. **(A)** Taste value; **(B)** Hardness; **(C)** Stickiness; **(D)** Peak viscosity; **(E)** Hot viscosity; **(F)** Final viscosity; **(G)** Breakdown; **(H)** Setback; **(I)** Pasting temperature; **(J)** Amylose content; **(K)** Amylopectin content/Amylose content; **(L)** Gel consistency.

As shown in **Figure 1**, among the treatments, WS-IBF treatment had the most significant improvement in the taste quality of the two varieties (**Figure 1A**), which increased the taste value by 1.1–5.7% compared with the other three treatments. The stickiness (**Figure 1C**) of WS treatments were higher than those of NS treatments under the same N fertilizer ratio, and the taste value and stickiness gradually increased with increasing basal N fertilizer ratio under the same straw treatment. The hardness (**Figure 1B**) showed the opposite change.

Under the same N fertilizer ratio, WS treatments had no significant effect on hot viscosity (**Figure 1E**), final viscosity (**Figure 1F**) and pasting temperature (**Figure 1I**) but significantly reduced the setback value (**Figure 1H**), while the peak viscosity (**Figure 1D**) and breakdown value (**Figure 1G**) were increased by 5.8–14.6% and 2.6–17.4%, respectively. Under the NS and WS treatments, the peak viscosity, hot viscosity, final viscosity and breakdown value gradually increased with the increase of the ratio of basal N fertilizer; in contrast, the setback value and pasting temperature decreased.

**Figure 2:**
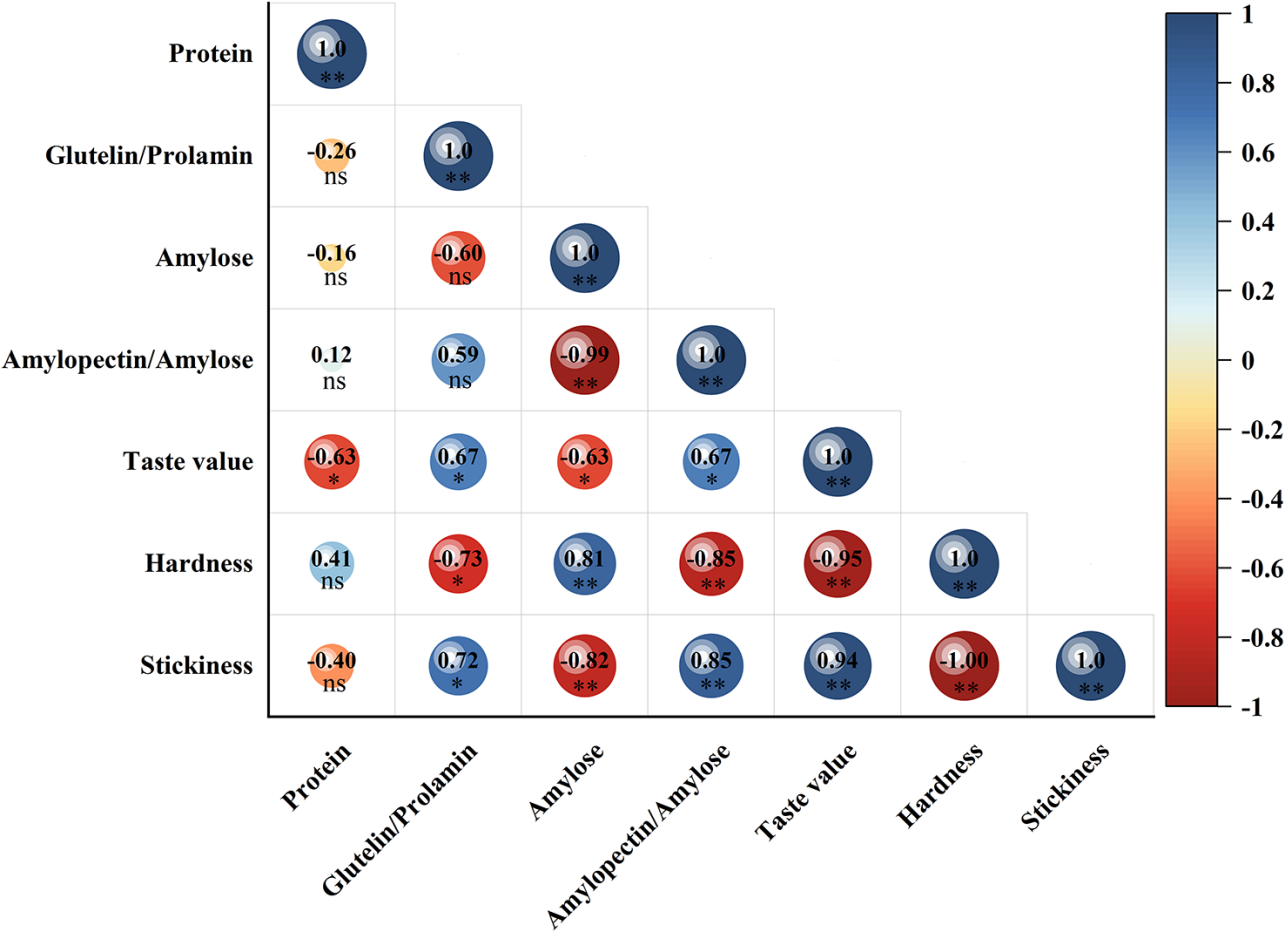
Correlations analysis between protein components, amylase content, ratio of amylopectin content to amylose content and cooking taste index. Note: *: significant at p < 0.05 level; **: significant at p < 0.01 level; NS, not significant at p < 0.05 level.

WS treatments reduced the amylose content (**Figure 1J**) and gel consistency (**Figure 1L**) of grain and significantly increased the ratio of amylopectin content to amylose content (**Figure 1K**) by 8.3–24.8%. Analysis-of-variance of F-values (**Table S3**) indicated that all the quality measurements showed significant difference (P<0.01) among the cultivars and treatments. Correlation analysis (**Figure 2**) showed that the amylose content had a significant negative correlation with taste value and stickiness and a significant positive correlation with hardness. The ratio of amylopectin content to amylose content was significantly positively correlated with taste value and stickiness and significantly negatively correlated with hardness.

### 3.5 Protein content and components in grains

WS treatments significantly increased the content of protein and its components under the same N fertilizer ratio compared with NS treatments (**Table 3**). Under the NS and WS treatments, protein content gradually decreased with the increase of basal N fertilizer ratio. Still, it increased the percentage of glutenin to prolamin by 0.9–3.0% for the two varieties. The protein, albumin, globulin, prolamin and glutenin content of NJ9108 were generally lower than that of HD5, but the ratio of high-quality glutenin to prolamin, which is not easily absorbed by the human body, was higher than HD5. Correlation analysis (**Figure 2**) indicated that protein content was significantly negatively correlated with taste value, the ratio of glutenin content to prolamin content had a significant positive correlation with taste value and stickiness, and a significant negative correlation with hardness.

**Table 3:** Protein content and its components in grains.

Time	Cultivar	Treatment	Protein (%)	Albumin (%)	Globulin (%)	Prolamin (%)	Glutelin (%)	Other Proteins (%)	Glutelin/ Prolamin
2020	NJ9108	NS-LFP	7.07 ± 0.18 c	2.93 ± 0.15 c	8.54 ± 0.08 ab	10.54 ± 0.10 b	35.27 ± 0.16 c	42.72 ± 0.18 b	3.35 ± 0.03 c
		NS-IBF	6.62 ± 0.14 d	2.58 ± 0.14 d	8.46 ± 0.12 b	10.26 ± 0.08 c	35.09 ± 0.05 c	43.61 ± 0.14 a	3.42 ± 0.03 a
		WS-LFP	8.12 ± 0.09 a	3.54 ± 0.08 a	8.62 ± 0.10 a	10.77 ± 0.12 a	36.47 ± 0.13 a	40.60 ± 0.23 d	3.39 ± 0.05 ab
		WS-IBF	7.45 ± 0.09 b	3.24 ± 0.05 b	8.58 ± 0.13 ab	10.60 ± 0.10 b	36.19 ± 0.17 b	41.39 ± 0.11 c	3.42 ± 0.05 a
	HD5	NS-LFP	7.68 ± 0.10 c	3.15 ± 0.05 b	8.70 ± 0.16 c	10.74 ± 0.12 b	35.60 ± 0.26 c	41.81 ± 0.18 b	3.31 ± 0.02 b
		NS-IBF	7.42 ± 0.13 d	2.98 ± 0.11 c	8.44 ± 0.13 d	10.51 ± 0.14 c	35.20 ± 0.23 d	42.87 ± 0.17 a	3.35 ± 0.04 ab
		WS-LFP	8.57 ± 0.12 a	3.44 ± 0.08 a	9.39 ± 0.14 a	11.21 ± 0.09 a	37.44 ± 0.11 a	38.52 ± 0.08 d	3.34 ± 0.03 ab
		WS-IBF	8.38 ± 0.07 b	3.20 ± 0.05 b	8.96 ± 0.18 b	10.90 ± 0.15 b	36.98 ± 0.12 b	39.96 ± 0.18 c	3.40 ± 0.05 a
2021	NJ9108	NS-LFP	7.33 ± 0.06 c	2.84 ± 0.04 b	8.67 ± 0.11 bc	10.28 ± 0.08 b	35.31 ± 0.37 b	42.90 ± 0.48 b	3.44 ± 0.05 c
		NS-IBF	6.98 ± 0.08 d	2.64 ± 0.07 c	8.55 ± 0.10 c	9.83 ± 0.15 c	34.73 ± 0.15 c	44.25 ± 0.19 a	3.53 ± 0.06 a
		WS-LFP	8.04 ± 0.12 a	3.40 ± 0.06 a	8.86 ± 0.13 a	10.61 ± 0.12 a	36.71 ± 0.15 a	40.42 ± 0.12 d	3.46 ± 0.04 bc
		WS-IBF	7.79 ± 0.07 b	3.34 ± 0.04 a	8.76 ± 0.12 ab	10.38 ± 0.04 b	36.48 ± 0.09 a	41.04 ± 0.14 c	3.52 ± 0.02 ab
	HD5	NS-LFP	7.97 ± 0.18 b	2.96 ± 0.08 b	8.81 ± 0.13 b	10.95 ± 0.12 c	36.02 ± 0.13 b	41.26 ± 0.11 b	3.29 ± 0.03 c
		NS-IBF	7.34 ± 0.15 c	2.61 ± 0.13 c	8.61 ± 0.11 c	10.53 ± 0.16 d	35.23 ± 0.25 c	43.02 ± 0.20 a	3.39 ± 0.05 a
		WS-LFP	8.49 ± 0.10 a	3.25 ± 0.09 a	9.32 ± 0.04 a	11.30 ± 0.04 a	37.73 ± 0.23 a	38.40 ± 0.20 d	3.34 ± 0.02 b
		WS-IBF	8.27 ± 0.11 ab	3.10 ± 0.11 ab	9.17 ± 0.08 a	11.13 ± 0.05 b	37.49 ± 0.22 a	39.11 ± 0.25 c	3.37 ± 0.03 ab
Source of variation									
Year (Y)			22.368**	37.987**	28.270**	8.089**	37.143**	9.981**	31.929**
Cultivar (C)			597.146**	NS	140.233**	495.432**	355.001**	1285.038**	132.948**
Treatment (T)			488.770**	273.267**	86.376**	165.573**	582.007**	1801.219**	21.922**
Y×C			19.691**	28.220**	NS	80.552**	21.645**	24.400**	37.581**
Y×T			9.380**	4.570**	NS	4.590**	3.696*	23.649**	NS
C×T			5.003**	23.095**	28.437**	NS	10.738**	31.060**	NS
Y×C×T			7.905**	5.201**	3.018*	NS	4.215**	3.982*	NS

NS-LFP, wheat straw non-returning and the ratios of basal fertilizer: tiller fertilizer: panicle fertilizer=5:1:4; NS-IBF, wheat straw non-returning and the ratios of basal fertilizer: tiller fertilizer: panicle fertilizer=7:1:2; WS-LFP, wheat straw full returning and the ratios of basal fertilizer: tiller fertilizer: panicle fertilizer=5:1:4; WS-IBF, wheat straw full returning and the ratios of basal fertilizer: tiller fertilizer: panicle fertilizer=7:1:2. Different lowercase letters in the same column indicate statistical significance at P < 0.05 in the same variety in the same year. * and ** significant at P < 0.05 and P < 0.01, respectively; NS, nonsignificant at P < 0.05 level.

### 3.6 2-AP contents

**Figure 3:**
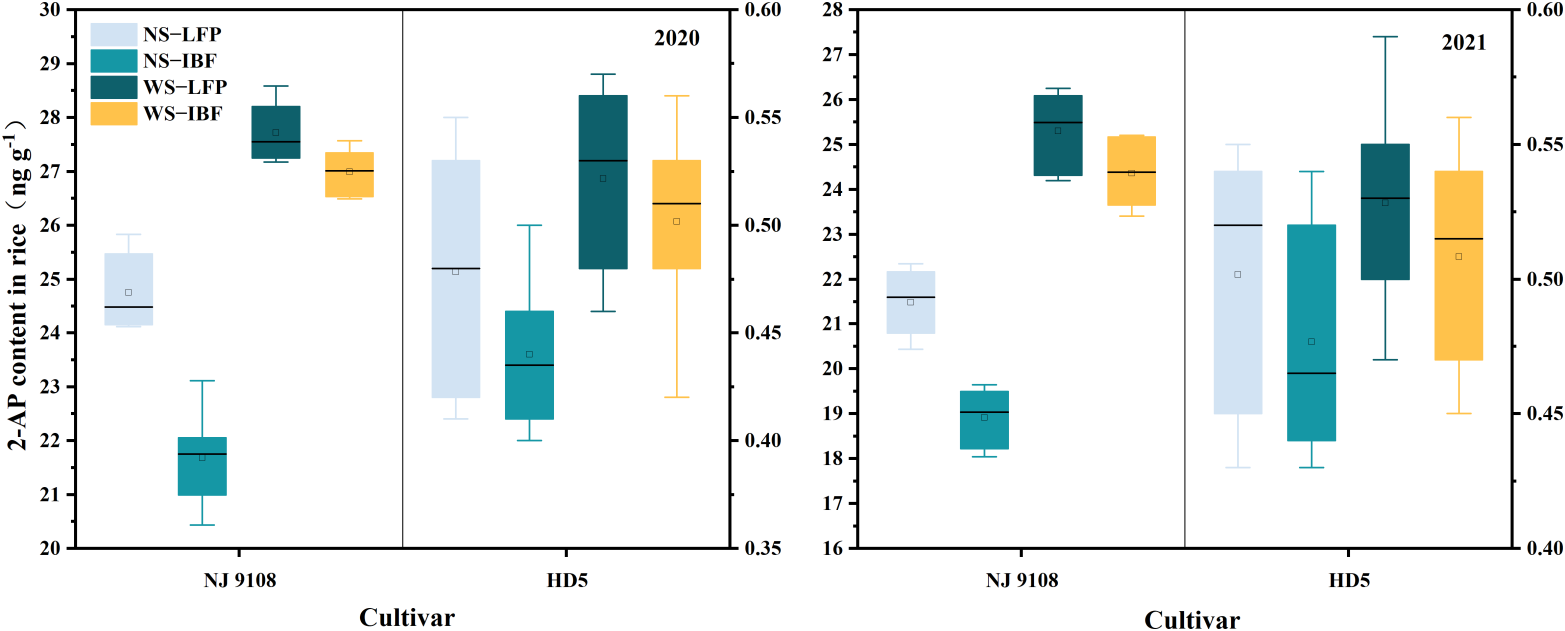
2-Acetyl-1-Pyrroline(2-AP) content in milled rice. Note: The left axis is the content of 2-AP in NJ 9108, and the right is the content of 2-AP in HD5.

There is a significant difference in the 2-AP content of milled rice between the two varieties (**Figure 3**). The 2-AP content of fragrant rice variety NJ9108 is between 18.4–27.7 ng g^-1^ and the content detected for non-fragrant rice variety HD5 is between 0.4–0.5 ng g^-1^. Under the same N fertilizer ratio, WS-LFP treatment increased the 2-AP content of NJ9108 by 12.0–17.8% compared with NS-LFP treatment, while WS-IBF increased by 24.5–28.8% compared with NS-IBF treatment, which indicated that WS treatments had a more significant increase in 2-AP content under the IBF fertilizer ratio. Under NS and WS treatments, the 2-AP content in milled rice was decreased by increasing the basal N fertilizer ratio, and there was a significant difference among different N fertilizer ratios under the condition of NS treatments. Compared with NS-LFP treatment, NS-IBF treatment reduced the 2-AP content of NJ9108 by 12.0–12.4%, while WS-IBF treatment only reduced 2.6–3.7% compared with WS-LFP treatment.

### 3.7 Proline content and enzymes activities involved in the 2-AP synthesis

**Figure 4:**
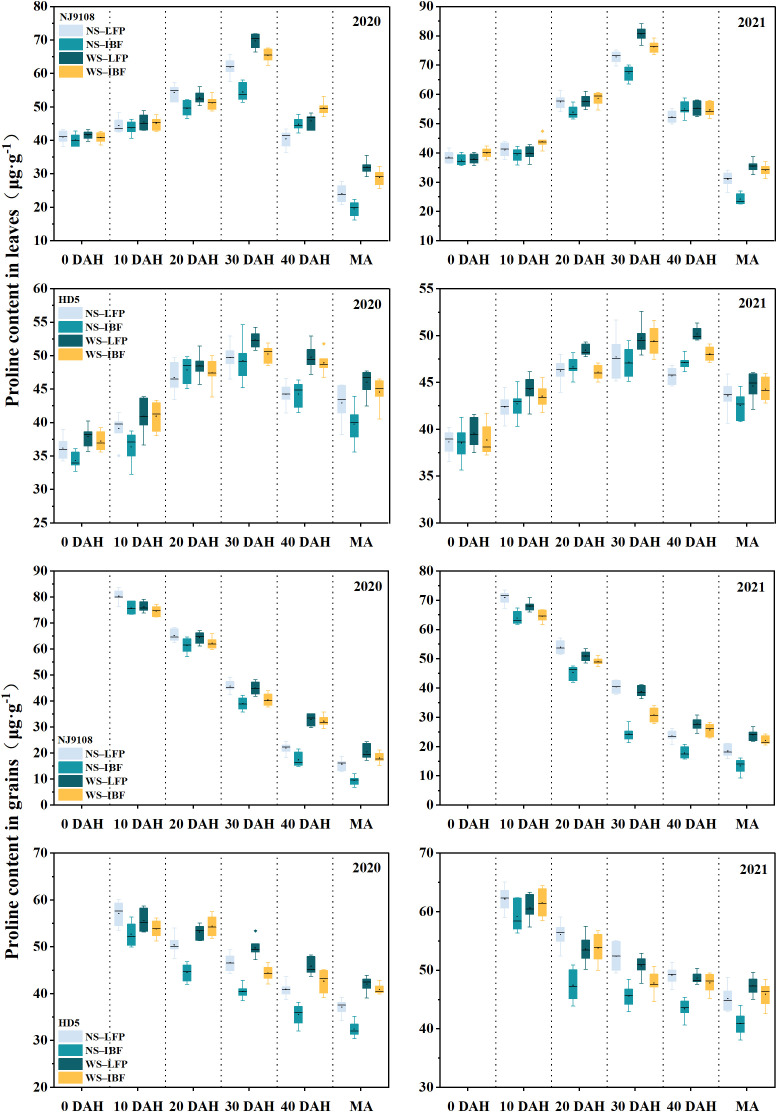
The proline content in leaves and grains from heading to maturity. Note: DAH, days after heading; MA, maturity.

With the increase of days after heading, the leaf proline content of NJ9108 and HD5 increased first and then decreased, reaching the highest value at 30 days after heading, and the grain proline content gradually decreased (**Figure 4**). The variation range of proline content of HD5 was smaller than that of NJ9108. Compared with NS treatments, WS treatments increased the proline content in the leaves and grains of the two varieties, and increasing the basal N fertilizer ratio would reduce the proline content in leaves and grains. However, there was no significant difference in grain proline content between WS-LFP treatment and WS-IBF treatment after 40 days of heading, while NS-IBF treatment decreased more than NS-LFP treatment.

**Figure 5:**
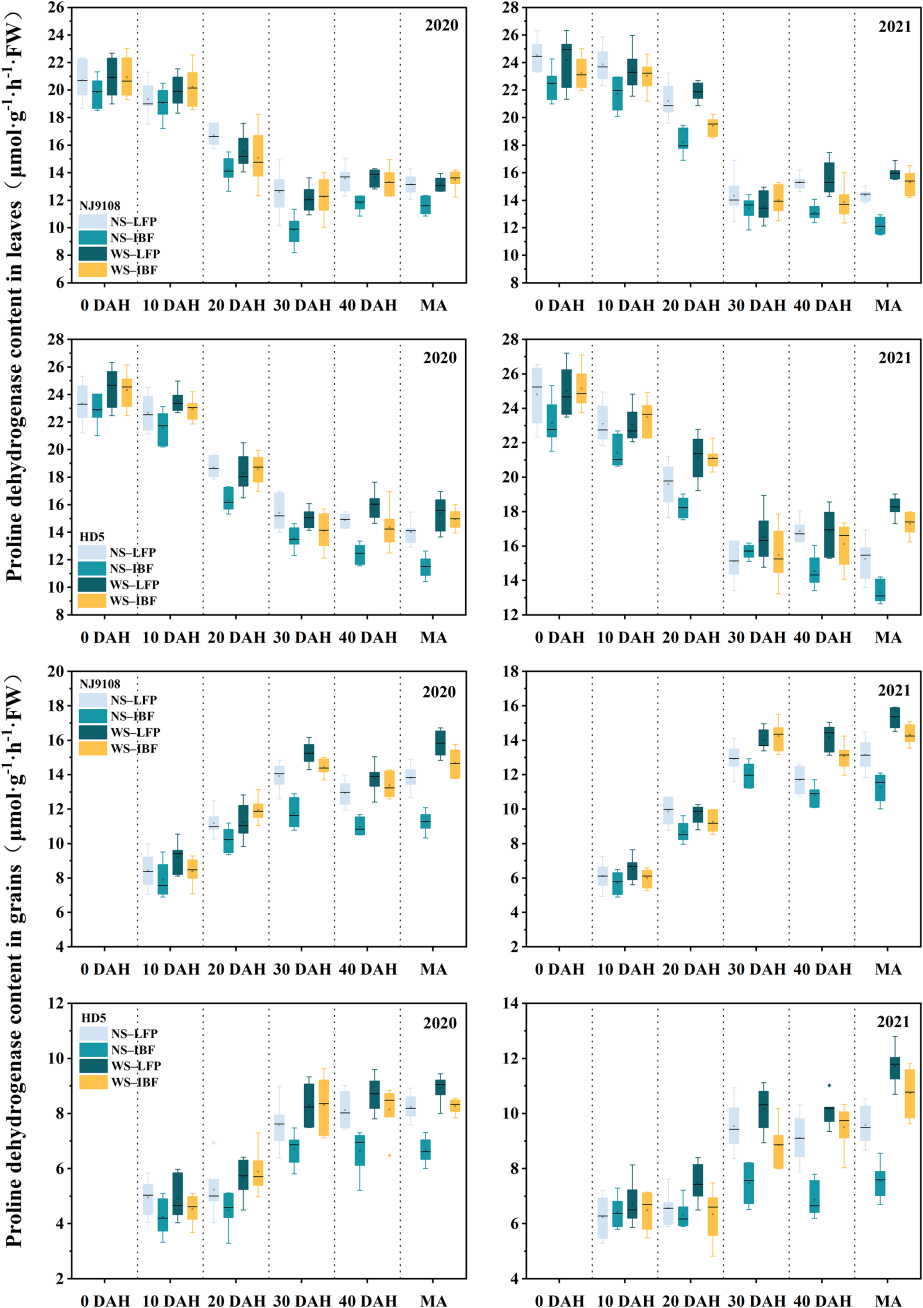
Activities of proline dehydrogenase (PDH) in leaves and grains from heading to maturity. Note: DAH, days after heading; MA, maturity.

**Figure 6:**
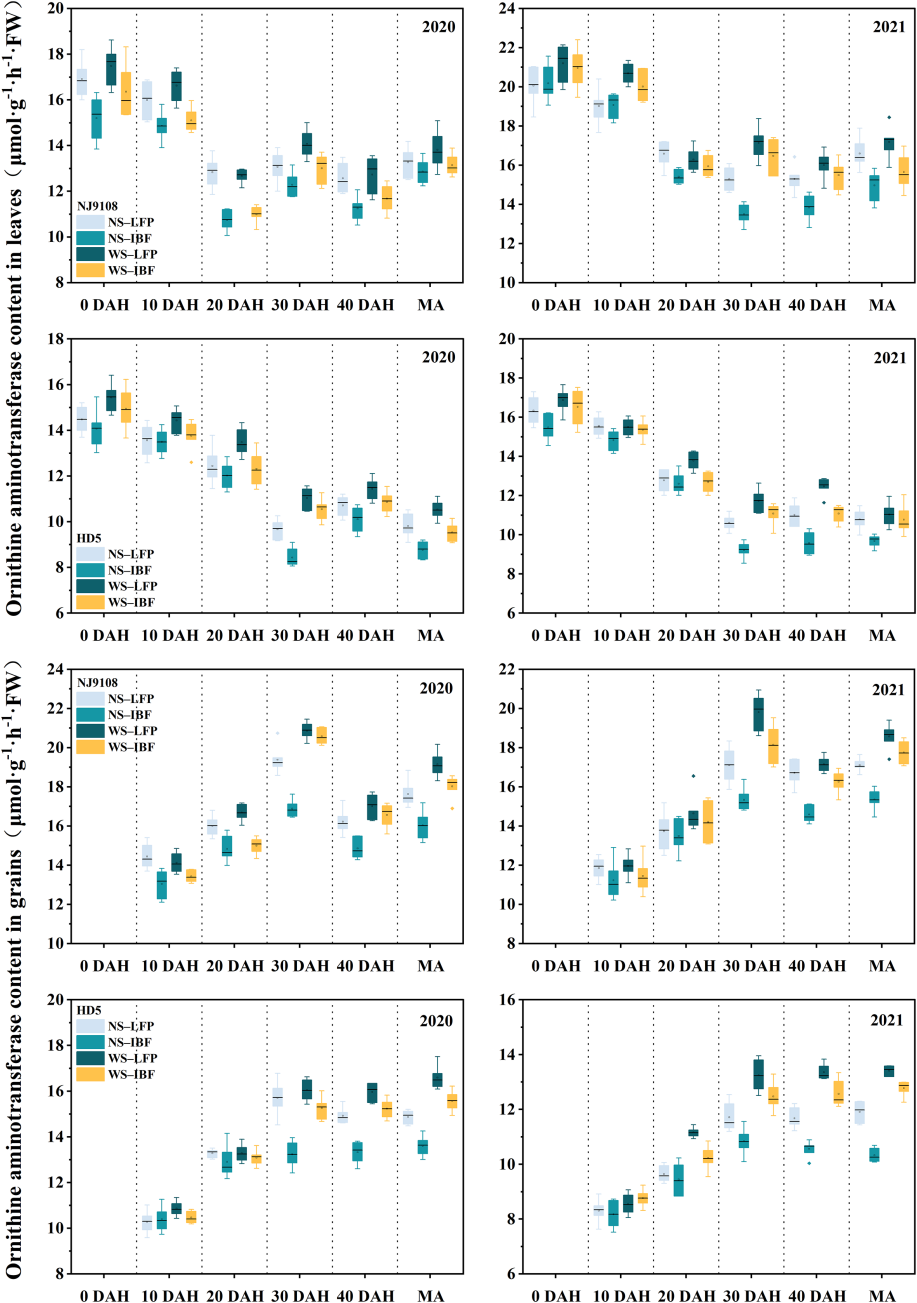
Activities of ornithine aminotransferase (OAT) in leaves and grains from heading to maturity. Note: DAH, days after heading; MA, maturity.

The activities of proline dehydrogenase (PDH) (**Figure 5**) and ornithine aminotransferase (OAT) (**Figure 6**) in leaves of the two varieties generally showed a downward trend from the heading stage. Except for the leave OAT activity of NJ9108, which increased slightly at 30 days after heading, the enzyme activities of the other treatments all decreased significantly from 0 to 30 days after heading. On the other hand, the enzyme activity changed slightly from 30 days after heading to maturity and increased slightly in some treatments. The activities of the two enzymes in grains increased gradually with the advancement of the growth period, and the changes tended to be flat after 30 days of heading. Under the same N fertilizer ratio, the enzyme activities of WS treatments were significantly higher than that of NS treatments. At maturity, WS treatments increased grain PDH enzyme and OAT enzyme activities by 14.9–34.7% and 8.6–19.1% compared with NS treatments. Under NS and WS treatments, increasing basal N fertilizer ratio reduced the enzyme activity, and NS-IBF treatment reduced the PDH enzyme and OAT enzyme activity of the two varieties by 16.4–20.1% and 9.7–10.9% at maturity compared with NS-LFP treatment. However, compared with WS-LFP treatment, WS-IBF treatment only reduced the activities of these two enzymes by 6.8–7.8% and 5.1–5.4%, and the difference was slight.

Correlation analysis (**Table 4**) showed that 2-AP content in milled rice at maturity was significantly positively correlated with proline content in grains and leaves 30 days after heading to maturity; It also had a significant positive correlation with the grain proline dehydrogenase activity 20 days after heading to maturity, and a significant positive correlation with the leave proline dehydrogenase activity at 40 days after heading; In addition, the 2-AP content in milled rice at maturity was also significantly positively correlated with grain ornithine aminotransferase activity 30 days after heading to maturity, and significantly positively correlated with leave ornithine aminotransferase activity 30-40 days after heading. The results showed that wheat straw returning was beneficial to increase the activities of proline dehydrogenase and ornithine aminotransferase in grains at the middle and late stage of grain filling, and increasing the proline content, the precursor of 2-AP synthesis, thus promoting 2-AP synthesis in grains.

**Table 4:** Correlations analysis between the 2-AP content in grains at maturity and other 2-AP-related physiological parameters.

	Position	0DAH	10DAH	20DAH	30DAH	40DAH	MA
Proline content	Grain	—	-0.167ns	0.200ns	0.501*	0.861**	0.889**
	Leaf	0.283ns	0.379ns	0.404ns	0.906**	0.432*	0.930**
PDH activity	Grain	—	0.404ns	0.513*	0.823**	0.780**	0.863**
	Leaf	0.276ns	0.323ns	0.105ns	0.403ns	0.506*	0.366ns
OAT activity	Grain	—	0.227ns	0.396ns	0.924**	0.801**	0.845**
	Leaf	0.400ns	0.293ns	0.334ns	0.650**	0.470*	0.341ns

*: significant at p < 0.05 level; **: significant at p < 0.01 level; ns, not significant at p < 0.05 level.

DAH, days after heading; MA, maturity

**Figure 7:**
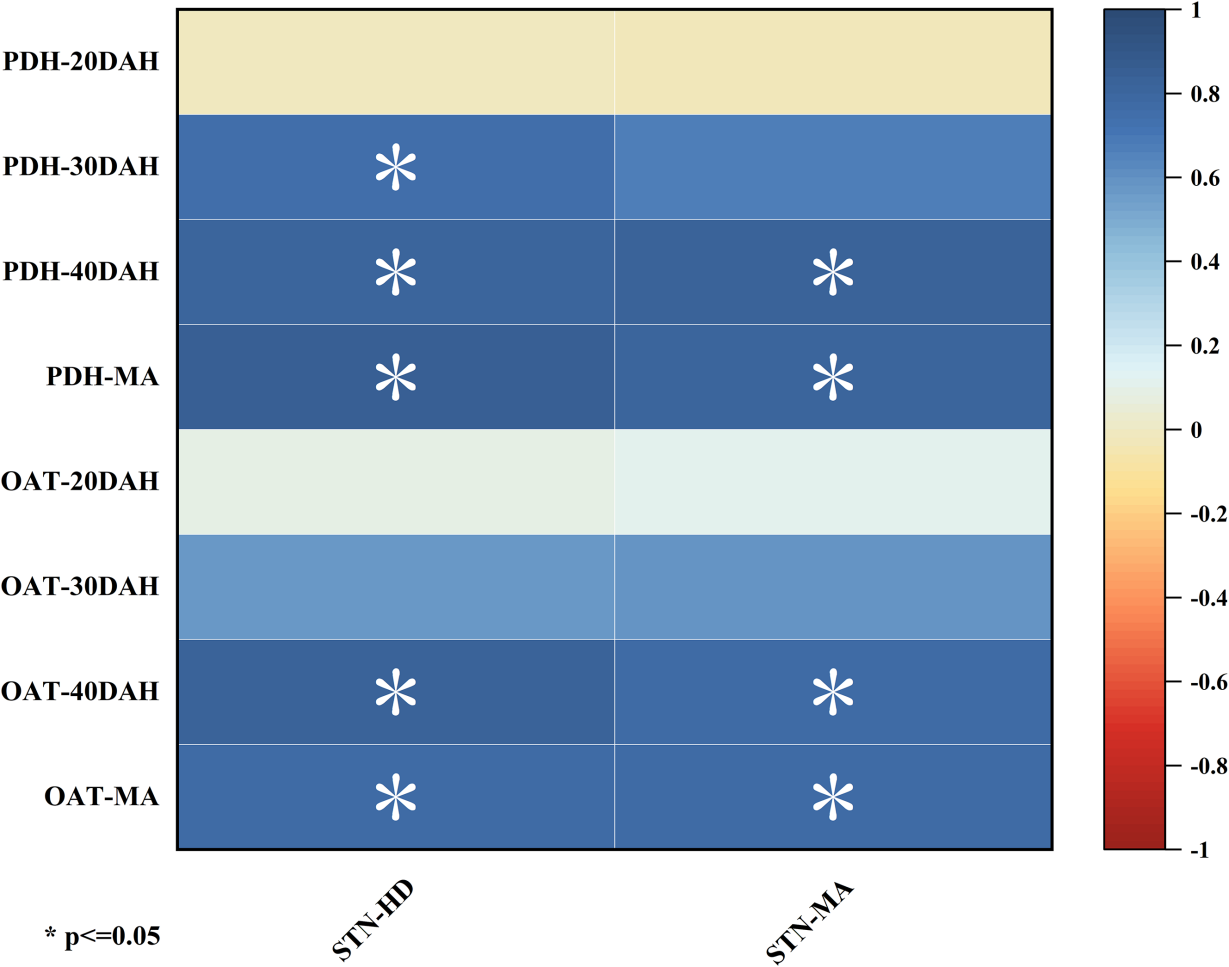
Correlations analysis between soil total nitrogen content at heading stage and maturity stage with enzyme activities of PDH and OAT in grains from 20 days after heading to maturity stage. Note: *: significant at p < 0.05 level. STN-HD, soil total nitrogen content at heading stage; STN-MA, soil total nitrogen content at maturity stage; PDH, proline dehydrogenase; OAT, ornithine aminotransferase; DAH, days after heading; MA, maturity.

Correlation analysis (**Figure 7**) showed that the soil TN content at heading stage was significantly correlated with PDH enzyme activity from 30 days after heading to maturity, and OAT enzyme activity from 40 days after heading and maturity; The content of soil TN at maturity was significantly correlated with the activities of PDH and OAT enzyme 40 days after heading and maturity.

## 4 Discussion

### 4.1 Coupling effects of wheat straw returning and basal N fertilizer rate on grain yield and dry matter weight

Straw returning is an effective way to improve soil properties and fertility. Many studies have shown that straw returning can improve the soil nutrient content and promote the uptake of soil nitrogen by roots, thus increasing the rice yield, and the yield increases with the increase of straw returning years (Wang et al., 2020; Cui et al., 2022). However, some studies held that straw returning has no significant effect on rice yield and even has a tendency to reduce it, which is related to soil texture and tillage methods (Zhu et al., 2011; Zhang et al., 2016). Kong et al. (2021) believed that the effect of straw returning on yield is closely related to the ratio between basal tiller fertilizer and panicle fertilizer. Straw is rich in a large number of carbon elements, and its C/N ratio is much higher than the suitable range of soil microorganisms. In addition, microorganisms will consume a certain amount of nitrogen during the decomposition of straw, which will lead to the reduction of nitrogen in the soil and fail to meet the normal growth of rice in the early stage (Tang et al., 2020). Pei et al. (2015) and Liu et al. (2017) deemed that wheat straw returning can reduce the occurrence of tillers in the early stage of rice and thus affect the yield, but significantly improve the growth indexes in the middle and late stages of rice, the results of this experiment were consistent with this. The panicle number of WS-LFP treatment was reduced compared with NS-LFP treatment under conventional N fertilizer ratio, while the yield increased slightly by a significant increase in the number of grains per panicle and the grain weight, but the difference between the two was not significant. Huang et al. (2013) and Tian et al. (2022) suggested that increasing the amount of N fertilizer used in the early stage under straw returning can reduce the inhibitory effect of straw returning on rice tillering so as to increase the number of panicles, and at the same time, the nitrogen released after straw decomposition delays the decline of leaf photosynthetic rate in the late growth stage of rice, which significantly improves the grain quality of superior and inferior grains, and eventually increases the yield. This study also showed that under wheat straw returning, appropriately increasing the basal N fertilizer ratio significantly enhanced the soil TN content thus augmenting the number of panicles and the grains per panicle, which greatly increased the yield. We also found that the panicle fertilizer rate in the WS-LFP was higher than that of WS-IBF, but the grain per panicle in the WS-LFP was lower than the WS-IBF, this is primarily due to IBF promoted straw decomposition and accelerated N release. We found that at the panicle-initiation stage, the increase rate of root dry weight of WS-IBF was higher than that of shoot dry weight compared with WS-LFP (**Table 2**). Liu et al. (2020) reported that increasing root dry weight at the panicle-initiation stage is conducive to promoting the spikelet differentiation and ultimately increase the grain per panicle.

Most studies have shown that the dry matter accumulation of rice in the early growth stage after straw returning to the field is smaller than that of the straw non-returning treatment (Pei et al., 2015; Li et al., 2017). This is mainly due to the accumulation of a large number of harmful substances, such as soil organic acids caused by straw returning; meanwhile, the need for nitrogen fixation by soil microorganisms to decompose the straw resulting in a phenomenon of competition for nitrogen between microorganisms and plants, so it will inhibit the accumulation of dry matter in the early stage of rice (Takakai et al., 2018; Zhang Y. J. et al., 2022). The results of this experiment are basically consistent with Pei et al. (2015) and Li et al. (2017). However, under the treatment with a higher basal N fertilizer rate, there was no significant difference in dry matter weight at the early growth stage between the WS treatments and the NS treatments, indicating that increasing the basal N fertilizer ratio under straw returning can alleviate the phenomenon of insufficient nitrogen supply in the early stage and reduce the inhibition effect of straw mulching (Yan et al., 2018). With the deepening of straw decomposition and nitrogen release in the later stage, the dry matter weights of all WS treatments were significantly higher than that of the NS treatments at the panicle-initiation stage, heading stage and maturity stage. Therefore, increasing the basal N fertilizer rate under wheat straw returning can effectively balance the population growth in the early and later stages of rice, significantly increase the dry matter weight of the rice population in the middle and later stages and ensure the coordinated growth of the number of panicles, the number of grains per panicle, and the grain weight, so as to achieve high yield.

### 4.2 Coupling effects of wheat straw returning and basal N fertilizer rate on physical and chemical characteristics of grain quality and protein components

Wu et al. (2022) concluded that wheat straw returning can significantly reduce the amylose content of rice, increase the gel consistency and improve the cooking quality of rice, and the results of this experiment are generally consistent with this. In this experiment, we found that under the LFP ratio, although wheat straw returning was beneficial to improve the stickiness and the ratio of amylopectin content to amylose content, meanwhile, reduce the hardness and amylose content of the grain, it also reduced the gel consistency and taste value, which tended to deteriorate the taste quality of rice, this is basically the same as the research results of Liu et al. (2007) and Hou et al. (2015). The effect of the N application ratio on rice cooking and eating quality was also inconsistent. Ju et al. (2018) showed that postponing nitrogen application can significantly improve the gel consistency of japonica rice, while Cheng et al. (2021) concluded that increasing N fertilizer at the later stage will increase the amylose content of rice, decrease the branching and crystallinity of amylopectin, and also the gel consistency. Hu et al. (2017) found that increasing basal N fertilizer lengthens the gel consistency, and the gel consistency of base tiller fertilizer: panicle fertilizer between 7:3 and 9:1 is significantly greater than that of 6:4. The starch RVA profile is also an important indicator of the cooking and eating quality of rice. Varieties with relatively high peak viscosity, breakdown values and low setback values are generally considered to have better taste quality. Most studies have shown that the peak viscosity and breakdown value of straw-returning treatment are higher than those of straw-non-returning treatment, while the setback value decreased (Ge et al., 2013; Chen et al., 2017), which can improve the palatability of rice. The results of this study are basically consistent with those of previous studies. Meanwhile, it was also found that the peak viscosity, hot viscosity, final viscosity and breakdown values of the WS-IBF treatment were higher than those of the other treatments, while the setback value was the opposite.

Grain protein is a key nutrient that determines rice nutrition and taste quality. In this study, the protein content of HD5 was higher than that of NJ9108 under all treatments, but the taste value of HD5 was significantly lower than that of NJ9108. Studies have shown that straw returning enhances the photosynthetic material production capacity and transport capacity of rice populations during the grain-filling period, and significantly increases the protein content of rice (Chen et al., 2014). Increasing the application of panicle and grain fertilizer can improve the activity of key enzymes of nitrogen metabolism in the rice grain filling stage, which is conducive to the degradation and transportation of leaf protein into the grain, increasing the content of protein and its components (Pan et al., 2010; Duan et al., 2013), thus reducing the rice taste quality. In this study, we found that WS-LFP treatment could reduce rice taste quality, mainly due to the significant increase in protein content. In contrast, WS-IBF treatment could reduce the content of protein and its components in grain, increase the ratio of glutelin to prolamin, and improve the taste quality and nutritional quality.

### 4.3 Coupling effects of wheat straw returning and basal N fertilizer rate on rice aroma and its physiological basis

Numerous studies have shown that the aroma of rice is not only controlled by genotype but also affected by soil, water, light, temperature and fertilizer (Goufo et al., 2010). There are few reports on the effects of straw returning on rice aroma, but it is generally believed that straw returning can enhance soil nitrogen levels, increase organic matter content and improve the soil environment, while soil with high organic matter and nitrogen is conducive to improving the activities of proline dehydrogenase and ornithine transaminase in rice grains, maintaining a high level of proline content in leaves and grains, thus promoting the increase of 2-AP content in grains at maturity (Yang et al., 2012), the results of this study are basically consistent with them. It has also been suggested that the decomposition of wheat straw in a flooded state causes the soil to behave in a strongly reducing state, resulting in the accumulation of hydrogen sulfide, organic acids, phenols and other substances, which have a stress effect on the growth of rice roots (Wang et al., 2015). As an osmoregulatory substance, proline can produce an active adaptive reaction in response to the stress and promote its synthesis and accumulation in plants to cope with stress damage (Liang et al., 2013). At the same time, straw decomposition releases a large amount of potassium, which is an important solute for cellular osmoregulation, and the increase in potassium promotes the activity of OAT, a key enzyme in the ornithine pathway of proline anabolism, further increasing the content of proline, a precursor substance for 2-AP synthesis (Cao and Wei, 2010; Zhang Z. et al., 2022). Correlation analysis showed that the content of 2-AP in grains at maturity was significantly or extremely significantly positively correlated with the precursor substances and related enzyme activities of 2-AP synthesis in grains at the middle and late filling stages. The effect of the N application ratio on rice aroma is also inconsistent. The research results of Mo et al. (2018) showed that increasing nitrogen fertilizer application at the booting stage can significantly increase the content of proline and 2-AP in plants, while Ren et al. (2017) believed that increasing nitrogen application at the tillering stage can promote leaf proline accumulation at early stage and increase the amount of proline translocated from leaves to grains at late growth stage of rice, so as to increase the 2-AP content in grains at maturity stage. On the contrary, some studies suggested that nitrogen application has adverse effects on the aroma of rice, and nitrogen content was negatively correlated with grain aroma content (Ganajaxi and Hegde, 2007; Jiang et al., 2015). In this study, under the NS and WS treatments, the 2-AP content, synthetic precursor substances and related enzyme activities decreased with the increase of basal nitrogen fertilizer rate. Under the condition of NS, the 2-AP content of grains at maturity stage in the NS-IBF treatment of NJ9108 decreased significantly, averaging 12.2% less than that in the NS-LFP treatment in two years, while under the condition of WS, WS-IBF treatment only decreased 3.2% compared with WS-LFP treatment, with no significant difference.

## 5 Conclusion

Wheat straw returning incorporated with higher basal nitrogen fertilizer (WS-IBF) was conducive to promoting the release of straw nitrogen, increasing the soil total nitrogen content and plant dry matter accumulation, thereby augmenting the number of panicles and grains per panicle, and ultimately improving the yield. WS-IBF could improve the taste value, peak viscosity, breakdown value, the ratios of amylopectin to amylose and glutelin to prolamin, reduce the content of protein and its components, setback value and amylose, thus improving rice taste and nutritional quality. The increase in soil total nitrogen content under wheat straw returning enhanced the activity of PDH and OAT, two key enzymes in glutamate and ornithine metabolic pathways in the middle and late stage, and increased the proline content, the precursor of 2-AP synthesis, thereby promoting the 2-AP accumulation in rice. Nevertheless, future studies should clarify the changes in soil physicochemical properties and microbial communities and their effects on 2-AP synthesis and accumulation in grains under wheat straw returning, and describe how these changes affect the genes upstream of PDH and OAT enzymes, so as to regulate changes in related enzyme activities to affect 2-AP synthesis.

## Data availability statement

The original contributions presented in the study are included in the article/**Supplementary Material**. Further inquiries can be directed to the corresponding author.

## Author contributions

JW and YQ performed the experiments and did the formal analysis and wrote the original draft, contributed equally to this work. XZ, ZZ, XH, YZ, LQ, KL, and SL assisted with the experiment. WW and YC revised and finalized the manuscript. JY and LL conceived and designed the experiment. All authors contributed to the article and approved the submitted version.

## Supplementary material

Supplementary Table 1 Tillering dynamics of rice Note: NS-LFP, wheat straw non-returning and the ratios of basal fertilizer: tiller fertilizer: panicle fertilizer=5:1:4; NS-IBF, wheat straw non-returning and the ratios of basal fertilize: tiller fertilizer: panicle fertilizer=7:1:2; WS-LFP, wheat straw full returning and the ratios of basal fertilizer: tiller fertilizer: panicle fertilizer=5:1:4; WS-IBF, wheat straw full returning and the ratios of basal fertilize: tiller fertilizer: panicle fertilizer=7:1:2. Different lowercase letters in the same column indicate statistical significance at P < 0.05 in the same variety in the same year. * and ** significant at P < 0.05 and P < 0.01, respectively; ns, nonsignificant at P < 0.05 level.

Supplementary Table 2 Soil total nitrogen content in different periods (g kg^-1^) Note: NS-LFP, wheat straw non-returning and the ratios of basal fertilizer: tiller fertilizer: panicle fertilizer=5:1:4; NS-IBF, wheat straw non-returning and the ratios of basal fertilize: tiller fertilizer: panicle fertilizer=7:1:2; WS-LFP, wheat straw full returning and the ratios of basal fertilizer: tiller fertilizer: panicle fertilizer=5:1:4; WS-IBF, wheat straw full returning and the ratios of basal fertilize: tiller fertilizer: panicle fertilizer=7:1:2. Different lowercase letters in the same column indicate statistical significance at P < 0.05 in the same variety in the same year. * and ** significant at P < 0.05 and P < 0.01, respectively; ns, nonsignificant at P < 0.05 level.

Supplementary Table 3 Analysis-of-variance of F-values of taste value, hardness, stickiness, peak viscosity, hot viscosity, final viscosity, breakdown, setback and pasting temperature between/among years, cultivars and treatments * and ** significant at P < 0.05 and P < 0.01, respectively; ns, nonsignificant at P < 0.05 level.
