# Supplementary material for: Increasing basal nitrogen fertilizer rate improves grain yield, quality and 2-acetyl-1-pyrroline in rice under wheat straw returning
Source: Front Plant Sci. 2023 Jan 12;13:1099751. doi: 10.3389/fpls.2022.1099751 (PMC9878184; doi:10.3389/fpls.2022.1099751)
Supplement: Supplementary Table 1 — Tillering dynamics of rice Note: NS-LFP, wheat straw non-returning and the ratios of basal fertilizer: tiller fertilizer: panicle fertilizer=5:1:4; NS-IBF, wheat straw non-returning and the ratios of basal fertilize: tiller fertilizer: panicle fertilizer=7:1:2; WS-LFP, wheat straw full returning and the ratios of basal fertilizer: tiller fertilizer: panicle fertilizer=5:1:4; WS-IBF, wheat straw full returning and the ratios of basal fertilize: tiller fertilizer: panicle fertilizer=7:1:2. Different lowercase letters in the same column indicate statistical significance at P < 0.05 in the same variety in the same year. * and ** significant at P < 0.05 and P < 0.01, respectively; ns, nonsignificant at P < 0.05 level. [file Table_1.docx]

| Time | Cultivar | Treatment | Tiller numbers (×10^4^ hm^–2^) | | | | Percentage of  productive tiller (%) |
| --- | --- | --- | --- | --- | --- | --- | --- |
|  |  |  | Mid-tillering | Panicle-initiation | Heading | Maturity |  |
| 2020 | NJ9108 | NS-LFP | 360.83±3.31 c | 385.17±3.43 c | 293.67±2.73 b | 290.50±2.88 c | 75.42±0.71 a |
|  |  | NS-IBF | 410.00±4.52 a | 424.00±2.37 a | 296.83±2.56 b | 296.50±4.04 b | 69.93±0.75 c |
|  |  | WS-LFP | 347.17±4.96 d | 379.17±3.76 d | 281.67±3.83 c | 280.67±3.67 d | 74.02±0.77 b |
|  |  | WS-IBF | 378.67±3.33 b | 401.33±4.37 b | 307.17±2.99 a | 307.00±3.69 a | 76.50±0.87 a |
|  | HD5 | NS-LFP | 357.83±1.72 c | 378.50±1.76 c | 282.33±3.20 c | 281.67±1.90 c | 74.42±0.57 b |
|  |  | NS-IBF | 398.00±1.90 a | 416.00±2.28 a | 289.17±2.32 b | 289.33±3.20 b | 69.55±0.69 d |
|  |  | WS-LFP | 343.83±2.86 d | 366.83±2.86 d | 269.83±2.93 d | 268.67±2.42 d | 73.24±0.71 c |
|  |  | WS-IBF | 371.17±3.13 b | 390.17±4.07 b | 296.50±2.17 a | 295.33±2.66 a | 75.70±0.89 a |
| 2021 | NJ9108 | NS-LFP | 356.00±4.56 c | 377.50±3.39 c | 287.17±2.48 b | 285.17±2.93 c | 75.54±0.74 b |
|  |  | NS-IBF | 390.67±3.33 a | 410.50±4.09 a | 291.67±4.13 b | 291.00±2.76 b | 70.90±1.04 d |
|  |  | WS-LFP | 341.83±2.93 d | 368.33±2.88 d | 271.33±3.56 c | 270.50±3.83 d | 73.26±0.83 c |
|  |  | WS-IBF | 372.17±4.02 b | 390.00±3.29 b | 302.50±3.08 a | 300.33±2.64 a | 77.01±0.77 a |
|  | HD5 | NS-LFP | 348.00±4.24 c | 374.67±2.94 c | 280.50±3.51 c | 278.00±2.68 c | 74.20±0.80 ab |
|  |  | NS-IBF | 379.50±2.74 a | 408.17±1.83 a | 286.67±2.88 b | 285.00±3.10 b | 69.83±0.79 c |
|  |  | WS-LFP | 331.13±3.88 d | 370.83±2.71 c | 272.67±3.20 d | 271.17±2.14 d | 73.13±0.91 b |
|  |  | WS-IBF | 366.67±2.73 b | 393.67±2.58 b | 294.17±2.32 a | 292.50±3.01 a | 74.31±1.08 a |
| Source of variation | | |  |  |  |  |  |
| Year (Y) | | | 202.366** | 86.244** | 37.670** | 56.410** | NS |
| Cultivar (C) | | | 113.831** | 52.802** | 146.593** | 147.711** | 37.892** |
| Treatment (T) | | | 1032.614** | 892.377** | 310.222** | 328.960** | 233.816** |
| Y×C | | | NS | 58.638** | 21.112** | 16.783** | NS |
| Y×T | | | 17.609** | 6.695** | NS | NS | NS |
| C×T | | | 3.581* | NS | 2.737* | NS | 2.894* |
| Y×C×T | | | NS | 5.268** | 4.177** | 5.405** | NS |

Table S1 Tillering dynamics of rice

Note: NS-LFP, wheat straw non-returning and the ratios of basal fertilizer: tiller fertilizer: panicle fertilizer=5:1:4; NS-IBF, wheat straw non-returning and the ratios of basal fertilize: tiller fertilizer: panicle fertilizer=7:1:2; WS-LFP, wheat straw full returning and the ratios of basal fertilizer: tiller fertilizer: panicle fertilizer=5:1:4; WS-IBF, wheat straw full returning and the ratios of basal fertilize: tiller fertilizer: panicle fertilizer=7:1:2. Different lowercase letters in the same column indicate statistical significance at P < 0.05 in the same variety in the same year. * and ** significant at P < 0.05 and P < 0.01, respectively; ns, nonsignificant at P < 0.05 level.

Table S2 Soil total nitrogen content in different periods (g kg^-1^)

| Time | Cultivar | Treatment | Mid-tillering | Panicle-initiation | Heading | Maturity |
| --- | --- | --- | --- | --- | --- | --- |
| 2020 | NJ9108 | NS-LFP | 1.35±0.04 bc | 1.67±0.03 b | 1.53±0.03 c | 1.38±0.03 b |
|  |  | NS-IBF | 1.39±0.03 ab | 1.57±0.05 c | 1.41±0.03 d | 1.34±0.04 c |
|  |  | WS-LFP | 1.33±0.04 c | 1.72±0.02 a | 1.63±0.04 a | 1.47±0.03 a |
|  |  | WS-IBF | 1.43±0.04 a | 1.75±0.04 a | 1.58±0.02 b | 1.44±0.02 a |
|  | HD5 | NS-LFP | 1.33±0.03 c | 1.63±0.03 b | 1.57±0.05 b | 1.42±0.03 b |
|  |  | NS-IBF | 1.41±0.04 b | 1.54±0.04 c | 1.47±0.02 c | 1.35±0.04 c |
|  |  | WS-LFP | 1.37±0.05 b | 1.75±0.04 a | 1.67±0.02 a | 1.52±0.02 a |
|  |  | WS-IBF | 1.55±0.03 a | 1.77±0.02 a | 1.60±0.03 b | 1.48±0.04 a |
| 2021 | NJ9108 | NS-LFP | 1.40±0.03 c | 1.70±0.06 b | 1.58±0.03 c | 1.41±0.02 c |
|  |  | NS-IBF | 1.46±0.03 b | 1.64±0.03 c | 1.52±0.03 d | 1.34±0.04 d |
|  |  | WS-LFP | 1.35±0.02 d | 1.74±0.04 ab | 1.69±0.02 a | 1.60±0.03 a |
|  |  | WS-IBF | 1.54±0.05 a | 1.76±0.03 a | 1.64±0.02 b | 1.52±0.02 b |
|  | HD5 | NS-LFP | 1.36±0.05 c | 1.68±0.04 c | 1.54±0.02 c | 1.41±0.04 b |
|  |  | NS-IBF | 1.45±0.04 b | 1.61±0.03 d | 1.48±0.03 d | 1.33±0.02 c |
|  |  | WS-LFP | 1.36±0.03 c | 1.76±0.03 b | 1.66±0.03 a | 1.52±0.02 a |
|  |  | WS-IBF | 1.52±0.04 a | 1.82±0.02 a | 1.59±0.04 b | 1.49±0.04 a |
| Source of variation | | |  |  |  |  |
| Year (Y) | | | 19.500** | 28.218** | 21.743** | 20.631** |
| Cultivar (C) | | | NS | NS | NS | NS |
| Treatment (T) | | | 94.236** | 130.720** | 184.196** | 179.960** |
| Y×C | | | 11.338** | NS | 45.365** | 25.811** |
| Y×T | | | NS | 3.047* | 2.931* | 6.289** |
| C×T | | | 5.344** | 7.107** | NS | NS |
| Y×C×T | | | 2.967* | NS | NS | 3.137* |

Note: NS-LFP, wheat straw non-returning and the ratios of basal fertilizer: tiller fertilizer: panicle fertilizer=5:1:4; NS-IBF, wheat straw non-returning and the ratios of basal fertilize: tiller fertilizer: panicle fertilizer=7:1:2; WS-LFP, wheat straw full returning and the ratios of basal fertilizer: tiller fertilizer: panicle fertilizer=5:1:4; WS-IBF, wheat straw full returning and the ratios of basal fertilize: tiller fertilizer: panicle fertilizer=7:1:2. Different lowercase letters in the same column indicate statistical significance at P < 0.05 in the same variety in the same year. * and ** significant at P < 0.05 and P < 0.01, respectively; ns, nonsignificant at P < 0.05 level.

| Source of variation | Taste  Value | Hardness | Stickiness | Peak  viscosity | Hot  viscosity | Final  viscosity | Breakdown | Setback | Pasting  temperature |
| --- | --- | --- | --- | --- | --- | --- | --- | --- | --- |
| Year (Y) | NS | 314.690** | NS | 172.946** | NS | 145.118** | 17.051** | 31.424** | 171.079** |
| Cultivar (C) | 1366.301** | 2727.877** | 1297.354** | 691.784** | 4938.579** | 917.233** | 2695.579** | 74.084** | 123.457** |
| Treatment (T) | 27.804** | 50.395** | 20.8498** | 377.272** | 151.168** | 109.153** | 22.275** | 143.402** | 37.891** |
| Y×C | 12.716** | NS | 38.464** | 84.605** | 7.466** | 26.486** | 7.744** | NS | 48.258** |
| Y×T | NS | NS | NS | NS | 7.145** | 10.041** | NS | NS | NS |
| C×T | NS | 6.279** | NS | 3.609* | NS | 9.834** | NS | NS | NS |
| Y×C×T | NS | NS | NS | 13.601** | 4.619** | NS | NS | NS | NS |

Table S3 Analysis-of-variance of F-values of taste value, hardness, stickiness, peak viscosity, hot viscosity, final viscosity, breakdown, setback and pasting temperature between/among years, cultivars and treatments

* and ** significant at P < 0.05 and P < 0.01, respectively; ns, nonsignificant at P < 0.05 level.
